# Energy and nutrient recovery from municipal and industrial waste and wastewater—a perspective

**DOI:** 10.1093/jimb/kuae040

**Published:** 2024-10-24

**Authors:** Lydia Rachbauer, Cesar B Granda, Shilva Shrestha, Werner Fuchs, Wolfgang Gabauer, Steven W Singer, Blake A Simmons, Meltem Urgun-Demirtas

**Affiliations:** Biological Systems and Engineering Division, Lawrence Berkeley National Laboratory, Berkeley, CA 94720, USA; BioVeritas, LLC, Bryan, TX 77807, USA; Department of Environmental Health and Engineering, Johns Hopkins University, Baltimore, MD 21218, USA; Department for Agrobiotechnology, Institute of Environmental Biotechnology, University of Natural Resources and Life Sciences, Vienna, 3430 Tulln, Austria; Department for Agrobiotechnology, Institute of Environmental Biotechnology, University of Natural Resources and Life Sciences, Vienna, 3430 Tulln, Austria; Biological Systems and Engineering Division, Lawrence Berkeley National Laboratory, Berkeley, CA 94720, USA; Biological Systems and Engineering Division, Lawrence Berkeley National Laboratory, Berkeley, CA 94720, USA; Joint Bioenergy Institute, Emeryville, CA 94608, USA; Argonne National Laboratory, Lemont, IL 60439, USA

**Keywords:** anaerobic digestion, biofilm, digestate treatment, carboxylate platform, mixed microbial community

## Abstract

This publication highlights the latest advancements in the field of energy and nutrient recovery from organics rich municipal and industrial waste and wastewater. Energy and carbon rich waste streams are multifaceted, including municipal solid waste, industrial waste, agricultural by-products and residues, beached or residual seaweed biomass from post-harvest processing, and food waste, and are valuable resources to overcome current limitations with sustainable feedstock supply chains for biorefining approaches. The emphasis will be on the most recent scientific progress in the area, including the development of new and innovative technologies, such as microbial processes and the role of biofilms for the degradation of organic pollutants in wastewater, as well as the production of biofuels and value-added products from organic waste and wastewater streams. The carboxylate platform, which employs microbiomes to produce mixed carboxylic acids through methane-arrested anaerobic digestion, is the focus as a new conversion technology. Nutrient recycling from conventional waste streams such as wastewater and digestate, and the energetic valorization of such streams will also be discussed. The selected technologies significantly contribute to advanced waste and wastewater treatment and support the recovery and utilization of carboxylic acids as the basis to produce many useful and valuable products, including food and feed preservatives, human and animal health supplements, solvents, plasticizers, lubricants, and even biofuels such as sustainable aviation fuel.

**One-Sentence Summary:**

Multifaceted waste streams as the basis for resource recovery are essential to achieve environmental sustainability in a circular economy, and require the development of next-generation waste treatment technologies leveraging a highly adaptive mixed microbial community approach to produce new biochemicals, biomaterials, and biofuels from carbon-rich organic waste streams.

## Graphical Abstract

**Graphical Abstract:**
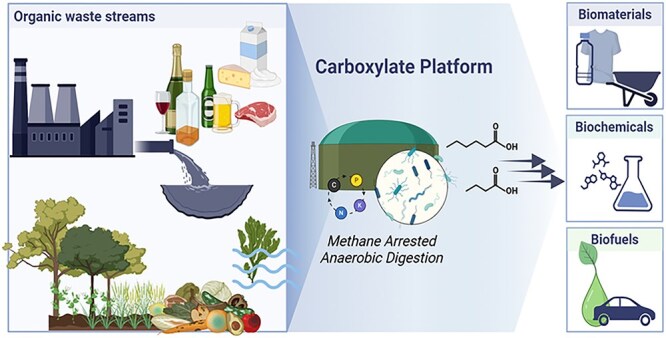

## Introduction

Reimagining our energy and carbon rich waste streams as a valuable resource can support overcoming current limitations with feedstock supply chains for biorefining approaches. Many current bio-based initiatives strongly rely on sugar-, lipid-, and starch-based input streams associated with land use change and the food/feed versus fuel debate. Extrapolations on how much carbon to build a future bioeconomy can be covered solely by sustainable biomass resources have their limitations. Although cascade utilization of the available biomass can counteract this limitation, the question remains where the necessary carbon should come from. A stepwise approach, where materials are the first priority, followed by chemicals production, and lastly fuel production, can enable a sustainable bioeconomy where the term ‘waste’ is redefined. Carbon waste streams are multifaceted and include municipal solid waste, industrial waste, agricultural by-products and residues, beached or residual seaweed biomass from post-harvest processing, and food waste. The food and beverage industry, including breweries, wineries, confectioners, and dairy producers, generates high-strength wastewater that requires treatment or costly disposal, varying by geography and quantity (Bochmann et al., 2020). Traditional treatment processes have low treatment efficiency or high operational costs and often ignore the economic potential of such carbon-rich waste streams. In a circular economy, resource recovery from waste streams is essential to achieve environmental sustainability. This requires the development of next-generation waste treatment technologies that produce new biochemicals, biomaterials, and biofuels from carbon-rich organic waste streams rather than simply disposing of them (Steinbusch et al., 2011; Tomás-Pejó et al., 2023).

On top of solid waste streams, gaseous carbon sources have attracted scientific interest. Point source streams, including industrial emissions from the steel mill industry, CO_2_ from biogas and bioethanol plants, as well as initiatives to concentrate CO_2_ via Direct-Air Capture open up a new space for a gas fermentation platform. Gasification of various types of biomass allows for syngas production, introducing a new technology on its way to becoming a major contributor to the future bioeconomy. Companies like Lanzatech, its spinoff LanzaJet, Synata BIO—who incorporated Coskata, Inc.’s syngas conversion technology, INEOS Bio, and JUPENG BIO, have been developing gas fermentation technology to convert synthesis gas from low-cost feedstocks into high-value products (Heijstra et al., 2017; Köpke and Simpson, 2020; Benevenuti et al., 2021; Liew et al., 2022). These examples demonstrate the feasibility of reimagining waste streams as a highly valuable resource.

Many current initiatives and future funding opportunities like the Sustainable Aviation Fuel (SAF) Grand Challenge Roadmap in the United States tackle challenges in sustainable fuel production and decarbonization efforts, particularly in hard-to-decarbonize sectors (Sustainable Aviation Fuel Grand Challenge Roadmap—Flight Plan for Sustainable Aviation Fuel, 2022). Developing a scalable, and robust bioconversion platform for carbon-rich waste streams contributes to advancing innovative energy technologies, facilitating the transition towards cleaner and more sustainable fuel production. Valorization of waste carbon streams into high-value and high-impact products as sustainable alternatives for the biomaterials, biochemicals, and biofuel sectors reduces environmental impact and promotes energy sustainability. In addition to reducing greenhouse gas emissions, bioconversion improves yield and energy efficiencies by using biogenic rather than fossil-derived inputs. This unique carbon benefit makes biomanufacturing at scale a more appealing alternative for producing most molecules, from the standpoint of CO_2_ emissions.

This perspective will highlight the latest advancements in the field of energy and nutrient recovery from municipal and industrial waste and wastewater. The emphasis will be on the most recent scientific progress in the area, the development of new and innovative technologies including microbial processes and the role of biofilms in the degradation of organic pollutants in wastewater, the production of biofuels and value-added products from wastewater and organic waste streams with a focus on carboxylic acids production via anaerobic digestion (AD). The authors acknowledge that other cutting-edge approaches such as gas fermentation strategies mentioned above will play a major role in the future circular economy that is based on waste carbon substrates. This highly important topic however, deserves in depth discussion elsewhere.

### Next-Generation Waste Treatment for Carbon-Rich Organic Waste Streams

Industries such as breweries, wineries, confectioners, slaughterhouses, renderers, and dairies generate voluminous amounts of high-strength wastewater that often require a tipping fee for disposal, varying by geography and quantity. For example, the production of 1 kg of cheese can generate 9–10 l of wastewater (Pires et al., 2021), and the production of 1 l of beer can generate 3–10 l of wastewater (Chen et al., 2016). The high chemical oxygen demand (COD) (15–110 g COD/l) of high-strength wastewater necessitates adequate treatment prior to disposal, but current waste treatment processes have low treatment efficiency or high operation costs. Resource recovery from high strength organic wastewater not only allows the extraction of value-added products and offsets the operational costs of wastewater treatment, but it is also conducive to alleviating adverse environmental issues.

The biodegradation of complex high-strength wastewaters requires a highly diverse microbial community structure. AD is an effective treatment process to convert large amounts of organic waste streams such as carbohydrate, protein, and lipid-rich wastewaters and food waste into high value renewable fuels (e.g., renewable methane) and products (e.g., volatile fatty acids) (Holtzapple et al., 2022). The mixed anaerobic microbial consortium or microbiome can have a significant biological diversity and syntrophic relationships, which enable the integration of multiple metabolic pathways from different kinds of microorganisms. Mixed microbial communities (MMC) are preferable to pure strains because the diversity of metabolic activities allows adaptation to varied operating conditions and complex feedstocks (Wu et al., 2021a).

Hydrolytic bacteria, acidogenic bacteria, acetogens, and methanogens are the main microbial communities responsible for AD of organic wastes. The first stage of AD is hydrolysis—complex and large organic molecules (proteins, carbohydrates, and lipids) are broken down into small compounds (amino acids, sugars, and long chain fatty acids) by microbes. In the second stage, acidogenesis, microorganisms convert the small molecules into volatile fatty acids, which are organic acids—also classified as short-chain (2–4 carbons) and medium-chain (5–8 carbons) carboxylic acids (acetic, propionic, butyric, valeric, caproic, heptanoic, and caprylic acids) (Holtzapple et al., 2022) along with other by-products. In the third stage (acetogenesis), the volatile fatty acids and other simple molecules created by the acidogenesis are converted into hydrogen, carbon dioxide, and acetic acid. In the final stage (methanogenesis), microorganisms convert the intermediate products of the preceding stages into methane, water, and carbon dioxide.

**Fig. 1.**
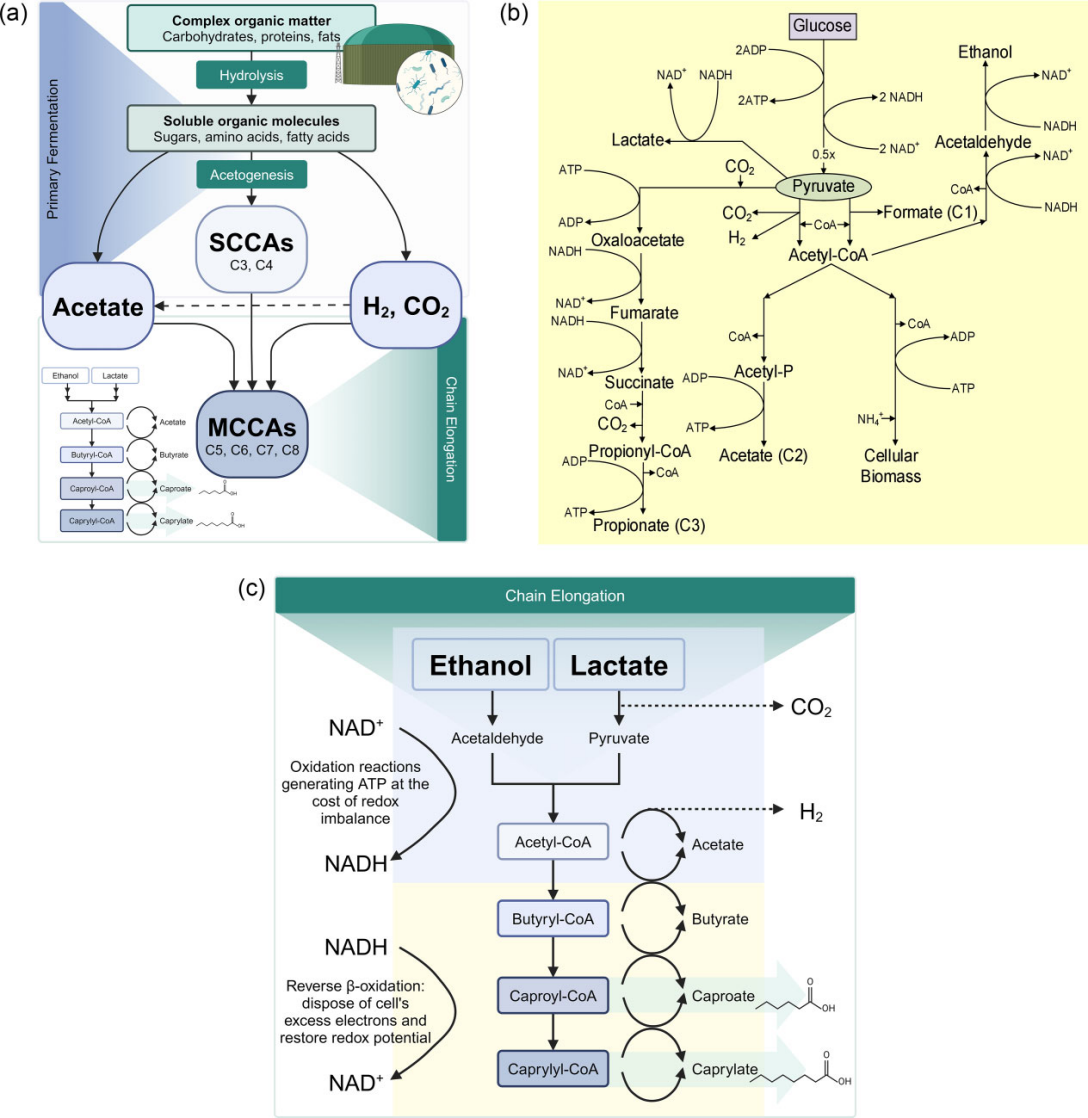
(a) Overall conversion observed in AM acidogenic fermentation. (b) Metabolic pathways in primary fermentation in AM acidogenic fermentation. (c) Metabolic pathways in secondary fermentation in AM acidogenic fermentation showing chain-elongation (reverse β-oxidation) as described in (De Groof et al., 2019). SCCAs, short-chain carboxylic acids; MCCAs, medium-chain carboxylic acids. Figure 1a (https://BioRender.com/l26u198) and c (https://BioRender.com/q31o230) were created using Biorender.

AD technology can be modified for the valorization of organic waste streams to produce short chain- and medium chain- carboxylic acids (SCCAs and MCCAs) consistently (Wu et al., 2021a). To this end, the AD process is rewired to produce carboxylic acids via arrested methanogenesis (AM), which is the basis for the carboxylate platform that can make a significant contribution to advanced waste and wastewater treatment (Holtzapple and Granda, 2009). To improve carboxylic acid production, the methanogens must be inhibited to avoid the consumption of SCCAs for methane production. The strategies inhibiting methanogens include inoculum pretreatment, a short sludge retention time and/or short hydraulic retention time, operation at low pH (pH <7.0), and the addition of chemical inhibitors. This modified AD process utilizes highly efficient, robust, and productive MMC structures for the conversion process. This MMC is an adaptable and stable ecosystem, which may not only adapt metabolically, but also microbially. The species present within the MMC fluctuate depending on the feedstocks, available nutrients, and operating conditions (e.g., temperature, pH, redox potential, etc). Such an MMC takes advantage of this diversity to efficiently convert any biodegradable material into carboxylic acids, ranging from acetic acid (C2) all the way to caprylic acid (C8). Figure 1a shows the different steps in the AM (or modified AD) conversion process, which starts with hydrolysis of complex molecules, such as carbohydrates, proteins and fats via enzymatic routes present in the MMC. The simpler molecules (sugars, amino acids, glycerol, and fatty acids) resulting from the hydrolysis step are then utilized by the same hydrolytic fermenters or syntrophically metabolized to SCCAs or short-chain fatty acids (formic, acetic, propionic, and butyric acid—C1, C2, C3, and C4), as well as hydrogen and CO_2_ (and ammonia in the case of proteins) in primary fermentation. Certain other intermediates such as alcohols (e.g., ethanol), lactic acid and succinic acid, are also produced but immediately metabolized. The SCCAs from the primary fermentation may undergo secondary fermentation or chain elongation, where they are elongated into the MCCAs or medium-chain fatty acids (valeric, caproic, heptanoic, and caprylic acid—C5, C6, C7, and C8) using the metabolic pathway known as reverse β-oxidation, by organisms affiliated with the *Clostridiaceae* and *Veillonellaceae* families, mainly in the *Clostridium* and *Megasphaera* genus (De Groof et al., 2019; Candry and Ganigué, 2021). Several metabolic pathways are at play during these transformations as seen in Fig. 1b and c. In Fig. 1b, primary fermentation pathways are depicted, showing the conversion of glucose into pyruvate as the initial step. From pyruvate most other products form, including cellular biomass and acetic (C2) acid, with an acetyl-CoA intermediate, alongside other SCCAs, namely formate (C1), and propionate (C3). In addition, other intermediates such as lactate and ethanol may play a crucial role in secondary fermentations (Fig. 1c) as electron donors for reverse β-oxidation. The oxidation of such electron donors to acetyl-CoA is coupled to the reductive elongation with SCCAs to form MCCAs, that is, butyric (C4), caproic (C6), and caprylic (C8) acids by adding two carbons in each step. Similar reverse β-oxidation can be observed with propionyl-CoA to form the odd-numbered MCCAs, valeric (C5) and heptanoic (C7) acids also by adding two carbons sequentially. In the anaerobic environment, these reactions occur under strongly reducing conditions, both primary and secondary fermentation are needed to provide sufficient free energy to generate ATP and restore NAD+/NADH balance in the cells.

Although ethanol-consuming chain elongation occurs in a variety of natural environments (including animal faeces and anaerobic digesters), the metabolic differentiation of chain elongators is widely unexplored as the microbial diversity seems to differ significantly (Candry et al., 2020). In addition to previously described *Clostridiaceae* and *Veillonellaceae* involved in chain elongation via reverse β-oxidation, a metatranscriptomic study on MCCA formation from lignocellulosic ethanol fermentation conversion residue identified a number of other potential chain elongators (Scarborough et al., 2018). Not previously associated with reverse β-oxidation, *Lachnospiraceae*- and *Eubacteriaceae*-affiliated organisms were predicted to transform primary fermentation products (acetate and lactate) to MCCA. These were the only two families in the metagenome-assembled genomes that contained genes encoding homologues of enzymes known to catalyze chain elongation reactions in the reverse β-oxidation pathway, thus thought to be responsible for MCCA production in this specific microbiome.

Using novel AM technology and associated MCCA producing MMCs, high concentrations of organic acids (35–78 g/l) from waste streams were produced at bench-scale digesters ranging from 0.5 to 14 l (Wu et al., 2021a, 2023, 2024). These values were the highest acid titers reported for organic waste streams in the literature. The most promising conditions were tested to scale-up the process in a 100-gallon digester (Wu et al., 2023). The pilot-scale results showed that the newly developed AM process has the potential for large-scale application and is an exemplary waste-to-energy technology that transforms low- or negative-value waste streams into high-value bioproducts. Experimental data also showed that SCCAs separation can make up to 64% of total SCCA production cost since their separation and purification require energy- and chemical-intensive separation processes with high capital and operating cost. A variety of low-cost and low-carbon intensity separation technologies (resins, membrane, and electrochemistry-based technologies) instead of distillation were tested to increase the product titer by 2–10 times and decrease the separation/purification costs by 75% (Wu et al., 2021b, 2024). High purity SCCAs can be used ‘as is’ or as a platform chemical for producing chemicals and fuels, such as SAF, with a low-carbon footprint.

This new waste treatment concept has the potential to disrupt the current waste treatment and management paradigm and can be readily integrated into current organic waste treatment and management practices. AM technology would eliminate the need for fossil-fuel derived feedstocks and introduce a responsible way to manage organic wastes and wastewater economically and environmentally friendly.

## Nutrient Recycling from Digestate

Due to the increasing demand for renewable energy and sustainable waste management, AD has become a widespread technology worldwide. This trend is particularly prominent in Europe, where ambitious targets have been set to diversify the energy mix (Lora Grando et al., 2017). Due to its high nutrient content, solid digestate from biogas plants fed with energy crops, organic waste streams, or farm manure is considered a high-quality fertilizer. The quality and quantity of the digestate produced are determined by the substrates used. Direct application of digestate to agricultural land remains the most common and cost-effective method (Fuchs and Drosg, 2010). The nutrients (nitrogen, phosphorus, potassium, and trace elements) in the biogas substrates can thus be returned to the agricultural areas where energy crops or food crops are cultivated. However, compliance with a maximum nutrient load per hectare and year is required, resulting in a corresponding area requirement for the application of biogas digestate. The high water content of digestate demands different treatment technologies to concentrate the nutrients and produce a transportable and marketable fertilizer (Kovačić et al., 2022).

The removal of suspended solids from digestate to purify the liquid fraction of digestate and allow nutrient recycling was evaluated previously (Meixner et al., 2015; Tambone et al., 2017; Beggio et al., 2022; Soja et al., 2023). A pilot test conducted at a biogas plant used a mobile decanter centrifuge (Flottweg C2E-4) for solid–liquid separation of the digestate. The aim was to concentrate the solids and produce a particle-free liquid fraction that could be further treated with membranes (nanofiltration and reverse osmosis) to concentrate the ammonium nitrogen and produce process water. Tests were conducted with varying amounts of flocculant polyacrylamide (PAM) and different g-forces of the decanter centrifuge. The best result was achieved using 8.05 kg of flocculant per ton of dry matter and a g-force of 3,350 g. The total undissolved solids were reduced from 8.15% solids in the original digestate to 1.98% in the liquid phase. The tests showed that a significant proportion of 75.70% of the solids could already be mechanically removed using a decanter centrifuge. However, this mechanical separation alone was insufficient to treat the liquid fraction directly with membranes. The total removal of fine particles was identified as a bottleneck in the treatment of digestate. Flocculation tests were conducted on different digestates from biogas plants using energy crops, manure, and organic waste to better understand the flocculation process and optimize the removal of fine particles. Jar tests were performed with different concentrations of FeCl_3_ (as the main flocculant) in combination with various polymers (e.g., PAM as auxiliary flocculant). To achieve a significant reduction in fine particles in the digestates, high amounts between 4.00% and 8.00% of Fe and between 2.00% and 4.00% of polymer were used relative to the dry matter content of the digestate.

The synthetic polymer PAM can have a very high molecular weight due to its synthesis and is the basis for efficient flocculants. There are concerns about the discharge of PAM with biogas digestate into the environment alongside the risk that acrylamide residues remain in the flocculants when PAM is synthesized (Lee et al., 2014). Biopolymers, such as chitosan which is derived from chitinous sources by either chemical or enzymatic treatment, represent a potential alternative for conventional PAM based flocculants. The following chitosan products were tested to flocculate biogas digestate and anaerobic, digested wastewater sludge at a dosage of chitosan and PAM of 4.00% of dry matter content: medium molecular weight, high molecular weight, acid-soluble, water-soluble, quaternary chitosan, and Biolog-Heppe (Chitosan 85/1000/A1) represent cationic chitosan products, while N-succinyl chitosan, and carboxymethyl chitosan exhibit a negative charge. Particle and flocculant charges are expressed by the zeta potential, an electrical potential that builds up on the particle surface of colloidal particles. This electric potential acts on other particles and exerts a force on them, which is the reason that ions can accumulate on the surface of the charged colloid in the so-called Helmholtz layer. As soon as the particle starts to move, this layer is sheared off and the particles show a charge. The zeta potential determines the magnitude of repulsion between the similar charged particles. (Shammas, 2005)

Since most of the colloids in the biogas digestate and anaerobic, digested wastewater sludge are negatively charged, the particles are repelled by their negative charges and remain in suspension for long periods of time. Fine particle distribution was measured (Particle Track G400 Mettler Toledo) after the jar-test and centrifugation in the supernatant. For the tested anaerobic, digested wastewater sludge, centrifugation alone without the addition of flocculant already reduced the original particle sum by 92.75% to ∼2,500 particles. Compared to PAM (Donau Multifloc 1023 M) as reference flocculent, the cationic chitosan products showed 20–40 times better results for residual fine particles sum for anaerobic, digested wastewater sludge after centrifugation (Table 1). However, the negatively charged N-succinyl chitosan and carboxymethyl chitosan showed no effect. This is thought to stem from the strong negative zeta potential of these polymers which results in repulsion from the also negatively charged particles in the sludge (Table 1). For biogas digestate, only a very low particle reduction for all tested chitosan products was achieved, whereas PAM significantly reduced the amount of fine particles in the biogas digestate. The significantly improved performance of PAM is likely linked to the high charge density for the commercially available PAM compared to the tested chitosan products.

**Table 1.** Zeta potentials, particle sums, and residual fine particle fractions of tested digestate and wastewater sludge, with tested chitosan flocculants (including PAM as reference material)

Substrate/flocculant	Zeta potential [mV]	Molecular weight [Da]	Initial particle sum	Residual particle sum after centrifugation
**Substrate**				
Biogas digestate	−36.5 ± 4.450	NA	–	–
Anaerobic, digested wastewater sludge	−23.5 ± 1.323	NA	33 675.7	2449
**Flocculant**				
Carboxymethyl chitosan	−48.7 ± 0.757	2 146	36 947.2	3003.6
N-succinyl chitosan	−45.6 ± 0.709	1 267	37 097.8	3909.8
Medium molecular weight chitosan	46.4 ± 0.321	93 294	42 393.3	23.0
Quaternary chitosan	47.4 ± 0.265	456 740	25 160.8	286.4
Acid-soluble chitosan	56.6 ± 0.265	334 445	35 048.1	54.9
High molecular weight chitosan	62.5 ± 1.739	476 749	37 472.9	20.3
Biolog-Heppe chitosan	63.8 ± 1.127	416 371	35576.0	10.8
Water-soluble chitosan	66.4 ± 0.889	537 684	37 779.2	15.5
PAM (Donau Multifloc 1023 M)	NA	NA	16 437.1	408.2

In addition to a generally high dry matter content in the digestate, a high proportion of fine particles and a high salt concentration in the digestate were identified as the most important adverse factors influencing flocculant efficiency. For biogas plants with a high dry matter content, a 2-stage solids removal process is recommended. This involves the use of a screw press, or a decanter centrifuge (without flocculants) followed by the treatment of the liquid phase with flocculants. Biogas plants using a high proportion of lignocellulose substrates, such as solid manure or straw, increase their fine particle content with the recirculation of digestate to dilute the main digester. To reduce the fine particle content and salt concentration, a portion of the recirculation volume can be replaced with water, or substrate fractions with very high salt concentrations can be identified and avoided, especially for waste biogas plants.

The direct application of untreated digestate to agricultural land as a high-quality fertilizer remains the preferred solution. However, if distances are too great, digestate processing technology must be employed to reduce volume and concentrate nutrients. Flocculant consumption can be minimized by adjusting the operating mode of the biogas plant, thereby altering the composition of the digestate. Additionally, further research is necessary to enhance the efficiency of alternative biodegradable polymers, such as modified starch or chitosan, with the ultimate goal of replacing PAM as a flocculant.

## Biofilm-Mediated Waste(Water) Treatment and Resource Recovery

Biofilms are cell aggregates that adhere to a surface or each other and are embedded in a matrix of extracellular polymeric substances (EPSs) containing a complex mixture of nucleic acids, carbohydrates, and proteins. Biofilm is prevalent in diverse natural and engineered systems and has attracted interest in multiple applications ranging from bioremediation, agriculture, food industries, medicine, wastewater treatment, and biomanufacturing. Conventionally, a biofilm-based approach has been used in wastewater treatment in rotating biological contactors and trickling filters for decades (Metcalf et al., 1991). Several bioreactor configurations including membrane bioreactors (MBRs), moving bed biofilm reactors, granular sludge-based systems, and integrated fixed film activated sludge take advantage of biofilm growth for wastewater treatment and resource recovery. Biofilm-based bioreactors can be used to intensify bioprocesses and improve performance due to high cell density and retention and high mass transfer for improved substrate utilization (Flemming et al., 2016; Philipp et al., 2023). Particularly, with the dynamic and complex composition of wastewater and waste streams, biofilm growth can provide a robust and resilient microbial community for efficient treatment. The EPS traps and concentrates nutrients making them readily available, and protects the microbial community from stressful external environmental conditions and inhibitory compounds typically present in waste(water). Additionally, high microbial diversity and the targeted gene expression controlled by quorum sensing allow the biofilm microbial communities to respond and adapt to the specific waste(water) characteristics ensuring efficient biodegradation (Sahreen et al., 2022; Li et al., 2023). In this context, biofilm-based systems can play an important role in wastewater treatment, especially for processes that rely on slow-growing microbes like anammox (Yuan et al., 2023), nitrifying bacteria (Wei et al., 2023), and anaerobes (Cayetano et al., 2022).

Biofilm systems are increasingly being developed for waste(water) treatment with simultaneous resource recovery. Particularly, MBR is a promising wastewater treatment technology due to its small footprint, the potential to produce high-quality and stable effluent, less sludge production, and uncoupling of the hydraulic and solid retention times among others. However, the MBR technology is still at the lab or pilot scales due to the high complexity compared to continuously stirred tank reactor technology and the high capital and operating cost due to membrane fouling. Dynamic membrane bioreactors (DMBRs) have emerged as an attractive alternative to traditional MBRs (Shrestha et al., 2021; Fairley-Wax et al., 2022; Fonoll et al., 2024). The DMBR takes advantage of the in-situ formation of the biofilm cake layer, also known as the dynamic membrane, on an inexpensive support membrane material to filter out particulates. Unlike conventional MBRs using microfiltration or ultrafiltration membranes, the use of cheaper membrane materials such as stainless steel mesh, nylon mesh, and carbon cloth, easier fouling control, and lower energy demand make DMBR an attractive alternative (Shrestha et al., 2021; Fairley-Wax et al., 2022; Samaei et al., 2023; Fonoll et al., 2024). While DMBR technology is still in its infancy, it has already shown wide application for waste(water) treatment with high COD removal and resource recovery using diverse substrates, including municipal wastewater (Jiao et al., 2022; Yang et al., 2023), high-strength synthetic wastewater (Fairley-Wax et al., 2022), brewery wastewater (Shrestha et al., 2021), waste-activated sludge (Kwon et al., 2023), and food waste (Cayetano et al., 2019; Fonoll et al., 2024). However, the suspended solids removal efficiency is not as high as a conventional polymeric membrane due to the larger pore size (10–200 µm) of DMBR membrane, often necessitating an additional filtration step when stringent water quality is required (for instance, water reclamation). Furthermore, more research needs to be done on the physicochemical and biological characterization of the dynamic membrane biofilm layer and develop strategies to achieve stable and long-term operation of DMBR.

The biofilm ecology including the microbial composition and structure, the effect of external conditions, and the EPS composition has been well-studied (Herschend et al., 2017; Shrestha et al., 2021; Candry et al., 2023). Due to substrate (nutrient) and oxygen gradient in the biofilm, there is localized niche separation allowing the growth of diverse microbial populations. The niche differentiation in the biofilm can be leveraged to spatially separate microbes with different physiology and achieve the desired function (Shahab et al., 2018, 2020; Jiang et al., 2023). Several studies have reported that biofilm growth led to higher microbial activity compared to planktonic growth leading to an increase in bioproduct formation such as lactic acid (Cuny et al., 2019), SCCAs (Xiros et al., 2019), and MCCAs (Shrestha et al., 2021). Similarly, bioelectrochemical systems also employ electroactive biofilm developed on electrodes for several applications including wastewater treatment and the production of biochemicals, bioplastics, and biofuels (Conners et al., 2022). There are still many opportunities and scientific and technological challenges to fully harness the potential benefits of biofilm for biotechnological applications.

Biofilm engineering is gaining attention to better control biofilm structure and dynamics and thus improve biofilm-mediated bioprocesses. Quorum sensing signaling molecules, second messengers such as c-di-GMP and cyclic AMP (cAMP), and small RNAs play important roles in regulating biofilm formation in several microorganisms and can serve as potential targets for biofilm engineering (Condinho et al., 2023). Several studies have demonstrated how altering the intracellular concentration of c-di-GMP or quorum sensing signals, exogenous addition of electron mediators such as quinones and flavins, and electrode modification in electrochemical systems can affect biofilm properties (Wood et al., 2016; Mukherjee et al., 2018; Li et al., 2023; Yi et al., 2023). Promoting cell adhesion to enhance biofilm formation in bioelectrochemical cells can improve current generation, while on the contrary, controlling the biofilm thickness can be useful to mitigate membrane biofouling in MBRs. However, engineering multi-species biofilms like those present in wastewater systems is not straightforward as it requires a detailed understanding of the pathways and regulatory system and the inter-species interactions associated with biofilm formation. Therefore, we need improved analytical methods to understand the complex structure, function, and regulation of biofilms and thus develop suitable targets and approaches for effective biofilm engineering.

## Energetic Valorization of High Strength Organic Rich Wastewater

Not only is material recovery from waste streams crucial, but energy recovery has also emerged as a pivotal issue in striving towards a sustainable, carbon-neutral society (Breach and Simonovic, 2018). Apart from harnessing the thermal energy content, the chemical energy stored in organic constituents can be leveraged. The theoretical potential is derived from the total COD, a parameter correlated to the sum of organic compounds in the effluent. Under the assumption of full extractability, the energy content is 13.9 kJ per g COD (Hao et al., 2019). Biogas formation via AD stands as the conventional and widely utilized method for energy recovery from wastes and wastewater (IEA, 2020). However, its efficiency heavily relies on the concentration of COD. Even with advanced reactor configurations, such as Internal Circulation reactors, COD levels above 2.0–3.0 g/l are requisite. A recent advancement is the anaerobic membrane bioreactor (AnMBR), where COD is concentrated through ultrafiltration membranes, enabling efficient treatment of low-strength wastewater (Mahmood et al., 2022). Such concepts are currently under investigation in a European-wide project termed SYMSITES (https://symsites.eu) and may allow the direct anaerobic treatment of municipal wastewater for biogas formation. Concerning the efficient utilization of generated biogas, there is a growing trend towards upgrading it to biomethane, suitable for injection into the gas grid, capitalizing on existing infrastructure (Ardolino et al., 2021). Microbial biogas upgrading, employing methanogenic archaea, is an emerging technique utilizing external H_2_ for converting CO_2_ into additional CH_4_. Previously reported results demonstrate the efficiency of this approach, capable of achieving quality standards for grid injection in a single unit operation (Rachbauer et al., 2016). However, in a future scenario where H_2_ replaces gaseous fuels, the next significant advancement is the formation of biohydrogen (bioH_2_) instead of biomethane. A currently feasible approach involves implementing a two-step process that divides AD into acidogenic and methanogenic reactors. During acidogenesis, a mixture of H_2_ and CO_2_ is produced, which can be separately collected (Luongo Malave’ et al., 2015). However, the H_2_ yields based on substrate input remain relatively low. Through collaboration with our research partners, various approaches have been explored to enhance bioH_2_ formation, including the use of thermophilic microorganisms (Ergal et al., 2018), synthetic microbial consortia (Ergal et al., 2022), or stimulating bioH_2_ production through low-voltage application (Hasibar et al., 2020). Even more sophisticated techniques aim at directly converting chemical energy into electricity using microbiological fuel cells (Munoz-Cupa et al., 2021). Therein, electro-active microbes catalyze redox reactions and transfer electrons directly to electrodes. Although this concept has garnered significant research interest and public attention, the complexity of reactor designs hinders practical implementation, and commercialization remains a long-term endeavor (Fuchs et al., 2023). An alternative approach to valorize chemical energy is the biotransformation of organics into extractable liquid energy carriers. Initial efforts focus on high-strength industrial waste streams or sludges from wastewater treatment. For instance, sugar or carbohydrate-rich effluents can be fermented to ethanol or butanol (Kundu et al., 2022). More recent concepts aim at simultaneous wastewater treatment and cultivation of oleaginous microorganisms, such as microalgae, bacteria, yeast, and filamentous fungi (Leong et al., 2019; Muller et al., 2014). Lipid accumulation in these organisms ranges from 20% to up to 80% (dry weight), showing promise as alternative feedstock in second-generation biodiesel production (Patel et al., 2020). In conclusion, a broad spectrum of technologies is either currently available or nearing practical implementation, poised to transform wastewater treatment from predominantly energy-intensive to a significant source of renewable energy.

## Recalcitrant Waste Streams: Bioconversion of Marine Seaweed as an Alternative Biorefinery Feedstock

Apart from industrial organic waste streams marine seaweed is another abundant, yet underutilized renewable resource for a future low to zero carbon bioeconomy. Marine seaweed has traditionally been used as food for human consumption, feed, and fertilizer, but provides a growing opportunity for renewable algal based biofuels and biochemicals. With an estimated annual production capacity of roughly 500 million dry metric tons in the USA, macroalgae could theoretically cover up to 10% the nation’s transportation energy demand (Mariner, ARPA-E, 2017).

While the market for whole seaweed biomass as food and food additive continues to grow (Cho et al., 2021), seaweed based biochemicals production is focused on single products (e.g., hydrocolloids, phenols, or colorants) (van Hal et al., 2014). For the hydrocolloid industry, mostly red and brown seaweed species serve as substrates to produce various grades of agar, alginate, and carrageenan. The less valued biomass at harvesting and leftover residues from post-harvest processing are considered waste. Whether seaweeds are sourced from cultivation (i.e., onshore and offshore farms) or wild collection, a significant amount of waste materials is generated during harvesting or such post-harvest processing steps (Yun et al., 2022). *Sargassum*, a prominent brown seaweed species, has gained increasing attention when it is washed to the tropical Atlantic shores in vast amounts, as beached or near-shore accumulations of *Sargassum* can have detrimental effects (Johns et al., 2020). Harvesting near-shore accumulating *Sargassum* biomass for biochemicals, feed, food, fertilizer, and fuel has been proposed as a potential solution to this seasonal problem (Milledge and Harvey, 2016). The complex polysaccharide structure of such seaweed entails unique properties that make these polymers highly attractive in biotechnological applications. However, it currently prevents its use as a feedstock for biomanufacturing and biofuels production as these polymers are different from those present in terrestrial feedstocks like ligno-cellulosic biomass, and are not directly compatible with existing technologies and process operations, suggesting that much more research is needed to elucidate the structure and associated degradation pathways (Laurens et al., 2020). Alginate, the main polysaccharide in *Sargassum*, makes up to 40.34% on a mass basis (Yudiati and Isnansetyo, 2017), and has dominated research on identifying novel alginate lyase enzymes (Yagi et al., 2016; Ghadam et al., 2017; Wang et al., 2017; Inoue, 2018; Pilgaard et al., 2021). Such alginate lyases are capable of breaking down the seaweed's recalcitrant polysaccharide structure to allow for alginate bioconversion to, for example, poly-3-hydroxybutyrate, a biodegradable polymer that is considered a sustainable plastic alternative (Moriya et al., 2020). Alternative approaches to enzymatic degradation such as seaweed ensiling and biogas production have been reported previously (Jard et al., 2013; Vanegas and Bartlett, 2013; Herrmann et al., 2015; Murphy et al., 2015; Ramirez, 2015; Milledge et al., 2019). AD under high salinity conditions as envisioned for a marine seaweed feed was reported to be beneficial to keep sulfur reducing bacteria (SRB) at bay. The presence of SRB is undesirable for the efficient production of methane, given that SRB compete with methanogens for hydrogen and acetate (Muyzer and Stams, 2008). Despite the high concentration of sulfates in a high salinity bioreactor, as expected for seawater, the proportion of SRB was lower in the high salinity bioreactor (35 ppt) compared to a low salinity bioreactor (10 ppt) (Derilus et al., 2019). Thus, a potential method of suppressing SRB numbers may be to modulate the salinity of the bioreactor, when saltwater is used. Certain seaweed species, however, are known to inhibit methane formation and have found a niche in preventing methane emissions, a very potent greenhouse gas, in cattle farming (Maia et al., 2016). *Asparagopsis taxiformis* is a type of red seaweed that has been found to reduce methane production in livestock rumen by 99% when ingested with everyday feed, at inclusion rates as low as 2% of total organic matter (Silwer, 2018). This effect could support an AM process as previously described and reroute the carbon flux into SCCAs instead of methane, effectively integrating the carboxylate platform into a seaweed biorefinery (Karunarathne and van Walsum, 2022). SCCAs such as acetic, propionic, butyric, and valeric acid, and the C6 molecule caproic acid have been reported to be biotechnologically produced through anaerobic fermentation via stable microbial communities from organic wastes of agricultural, industrial, or municipal origin (Tomás-Pejó et al., 2023). Marine seaweed remains a vastly underutilized resource with great potential to serve as an sustainable feedstock for biofuel and biochemical production, thereby wasting a huge opportunity of value addition to the biorefineries (Laurens and Nelson, 2020). Tapping into a conventionally inaccessible substrate like marine seaweed enables a scalable biomanufacturing process that utilizes the carbon contained in seaweed biomass. The anaerobic nature of such a system allows for easy scale-up at reduced energy requirement for oxygen supply, and reduced contamination risk when using a robust MMC which is adapted to high salinity compared to conventional fermentation systems using pure strains. The enhanced metabolic abilities within MMC enriched on *Sargassum* resulting from syntrophic interactions offer a promising potential for converting marine seaweed as low-cost feedstock into various bioproducts or biofuel (Derilus et al., 2019). Utilizing MMC for bioenergy and biofuel production from waste and residue streams is recognized as an environmentally sustainable strategy to reduce greenhouse gas emissions, decrease reliance on fossil fuels, promote sustainable waste management, and foster the transition to a circular economy.

## Industrial Perspective on the Carboxylate Platform to Treat and Upgrade Organic Effluents and Industry Low-Value by-Products

Although several wastes and byproducts, such as fat, oil, and grease, have found a niche in chemical conversion to biodiesel, renewable diesel or SAF, these wastes are limited and demand a high price (Zhang et al., 2020). Other forms of waste, such as food waste, sludge, animal manure, and dairy waste, on the other hand, can be obtained at a significantly lower cost with an order of magnitude greater availability (Badgett et al., 2019). The complexity and high moisture content of these waste feedstocks typically relegate their use to aerobic composting, wastewater treatment, or methane production by AD. The robustness and flexibility of traditional AD makes it a very advantageous process for processing these waste feedstocks. Beyond methanogenic biogas production via AD, it is well known, as discussed in previous sections, that intermediate carboxylic acids are also produced before being converted into methane and carbon dioxide. Such carboxylic acids demand a much higher price than methane and may be used as building blocks for the production of many useful products, which can serve as food and feed preservatives, human and animal health supplements, solvents, plasticizers, lubricants (Granda and Holtzapple, 2008; Jadhav et al., 2017; Atasoy et al., 2018; Ramos-Suarez et al., 2021), and even biofuels such as SAF (Huq et al., 2021). AM, therefore, is advantageous but requires adjusting or directing the mixed culture or consortium of microorganisms (MMC or microbiome) present in AD to inhibit methanogenesis and to preserve and enhance the production of such carboxylic acids. The production of mixed carboxylic acids by microbiomes is well-known and one of the most ancient fermentation processes, naturally occurring in the gastrointestinal tract of many animals, such as termites and the rumen of ruminants. In those environments, nature has evolved to make the process very efficient, obtaining very high conversion and yields of these carboxylic acids in just a few hours with even difficult-to-degrade complex feedstocks such as lignocellulosic biomass (Weimer et al., 2009). The process requires no sterile conditions, which has presented a significant opportunity for industrial bioconversion processes relying on sterile monocultures, which are typically plagued with high costs due to low culture stability, low flexibility, and high contamination issues. Towards its industrial deployment, the carboxylate platform (Holtzapple et al., 2022), which employs microbiomes to produce mixed carboxylic acids, and their recovery and utilization to produce many useful and valuable products, has been the subject of R&D and industrial development and implementation for many decades.

Production of mixed carboxylic acids at an industrial level began many centuries ago in the mud pits where Chinese Strong liquor (Baijiu) is manufactured. Grains, such as sorghum, are fermented into the World’s most consumed liquor. Many of these mud pits and their mixed cultures have been in continuous operation non-stop for centuries, with the oldest ones dating from the 16th century. They have a rich, stable microbiome and produce carboxylic acids, mainly caproic acid, which imparts a strong taste to the liquor and has been the subject of many studies (Zhu et al., 2015; Fu et al., 2021). Such a time-honored process attests to the stability and reliability of these MMC or microbiome-based fermentations for producing mixed carboxylic acids.

During World War I, with the demand for cordite (smokeless gun powder) and the shortage of acetone—the chemical required for cordite production—American businessmen created a new industry. California’s giant kelp, which grew extensively along the coast, was used as feedstock in an AM microbiome fermentation to produce calcium salts of mixed carboxylic acids, mainly acetate, which were then converted to acetone through a dry distillation or pyrolytic process. The complex nature of kelp as a seaweed, which contains complex carbohydrates such as alginate and mannitol, as mentioned in the section ‘Recalcitrant waste streams’, made AM microbiome fermentation particularly suited for the task (Neushul, 1989). Over 11 million pounds of acetone were made by this industry in the early 20th century. Further industry was established in France from the 1920s to early 1940s, with the company Le Ketol Société Anonyme founded by French engineer Louis Le Franc, who invented and patented a process that converted calcium salts of mixed carboxylic acids obtained from fermentation of sawdust into ketones, which were being commercialized as a solvent for industrial applications and a fuel with the name of Ketol (Le Franc, 1927, 1928). Both of these early 20th century commercial deployments of the carboxylate platform, in California and France, demonstrated the scalability and industrialization potential of the carboxylate platform.

Low oil prices put a halt to the carboxylate platform’s industrial implementation after World War II, but R&D by many different parties restarted during the oil shortages of the 1970s, with the aim of developing a low-cost bioconversion process that can inexpensively produce biofuels. Extensive research in the USA focused on the conversion of seaweed and other complex feedstocks to mixed carboxylic acids, with liquid-liquid extraction of the acids into a mix of kerosene and trioctyl phosphine, and Kolbe electrolysis of the acids into hydrocarbons (Levy et al., 1981). Playne (1980) in Australia focused on lignocellulosic biomass AM microbiome fermentation into mixed carboxylic acids with extraction by coupled membranes into calcium salts, which were then converted to ketones using pyrolysis. Datta (1981) in the USA, studied pretreated lignocellulosic biomass conversion to mixed carboxylic acids and recovery of the acids by conversion to esters. Others also performed R&D on mixed carboxylic acid production via AM microbiome fermentation and the carboxylate platform around the same time in other parts of the World, such as de la Torre & Goma (1981) in France.

With the climate change mitigation push in the 1990s, a renewed interest in biofuel production also took place. Research on AM microbiome fermentation for producing mixed carboxylic acids and the carboxylate platform occurred at Texas A&M University under Dr. Mark Holtzapple starting in the early 1990s (Holtzapple et al., 1999). The development continued throughout the 2000s (Holtzapple and Granda, 2009), with further innovations on the conversion of carboxylic acids to hydrocarbon fuels and extraction of the carboxylic acids. From this R&D, efforts to commercialize the carboxylate platform began in the mid 2000s, focusing on the conversion of mixed carboxylic acids to hydrocarbon biofuels. The company Terrabon, Inc. built a demonstration facility, with 30,000 gallons of fermentation capacity, which processed 3 tons of food waste per day into mixed carboxylic acid salts and further into hydrocarbon fuels, and operated stably for close to 2 years.

A strategy for deployment of the carboxylate platform would be targeting the chemical markets first for the carboxylic acids and derivatives, which demand a higher price compared to biofuels. Such a strategy could allow for smaller facilities to be constructed and de-risked, as a stepping stone towards a biofuels pipeline. BioVeritas, LLC has followed such a strategy for upcycling low-value byproducts or wastes from the agriculture and food sectors into high-value carboxylic acids for the chemical and food bio-ingredients markets. BioVeritas’ development has achieved the control of the acid profile in the AM microbiome fermentation by directing different conditions such as nutrients, pH, temperature, and other operating conditions, followed by an innovative technology for efficient extraction and recovery of the mixed carboxylic acids from the fermentation effluent.

Beyond chemicals, the next step in the carboxylate platform employing AM is the production of biofuels, such as SAF. The platform has demonstrated that biofuels can be effectively produced from organic wastes. However, scale-up will be critical to overcome the lower price point of biofuels compared to biochemicals, necessitating economies of scale. Additionally, the volumes required for biofuels are much larger, meaning production facilities must reach a certain size to make an impact and compete with incumbent fossil fuels. Ideally, the size of these facilities should align with ethanol fuel plants, which on average produce around 90 million gallons of ethanol per year, processing over 870 000 metric tons of corn annually (Ethanol Producer Magazine, n.d.).

Although the AM process may be de-risked through gradual scale-up using the high margin chemicals market as a stepping stone towards the low-cost biofuels sector, the main limitation remains the logistics of collecting and procuring waste feedstocks. The DOE’s 2023 Billion-Ton Report highlights the significant amount of distributed waste (71 million tons of waste generated annually on a dry basis, with substantial yearly growth, including animal manure, wastewater sludge, and food waste) (Langholtz, 2024). The report also emphasizes the logistical challenges of collecting such waste, which requires facilities to be located near the waste source, making it difficult to meet the yearly feedstock requirements for a centralized biofuel facility of desired scale. Developing efficient collection logistics is crucial to fully leverage these available wastes. Using established feedstocks, such as byproduct streams from the corn industry and a combination of agricultural residues, including lignocellulosic biomass, with wastes should also be considered as a potential strategy to improve feedstock procurement and logistics (Rughoonundun and Holtzapple, 2017).

In summary, tremendous progress has landed industrial innovators like BioVeritas on a steady path towards commercial deployment of the carboxylate platform within the next 3 years. It should be noted that widespread use of the carboxylate platform for waste feedstocks beyond chemicals into biofuels will require efforts towards improving waste collection and procurement logistics. Nevertheless, as new tools for improving understanding and controlling microbiomes and their interactions with feedstocks, nutrients, operating conditions, and the microbial community itself are becoming available (Lawson et al., 2019), the carboxylate platform anticipates a promising future poised for continuous improvement.

## Conclusion

This paper clearly outlines the vast potential of organic rich waste utilization in bioenergy, biofuels, and biochemicals as a cornerstone for sustainable development. Organic waste from the food and beverage industry, high-strength wastewater, and marine seaweed biomass are valuable resources from a bioeconomy standpoint. Utilizing MMC as present in AD or wastewater treatment systems for bioenergy, biochemicals, and biofuels production from organic waste streams has enormous potential to reduce greenhouse gas emissions, decrease reliance on fossil fuels, promote sustainable waste management, and foster the transition to a circular economy. Pilot-scale results showed that an AM process has the potential for large-scale application to manage high-strength wastewater economically and environmentally friendly. AM is an exemplary waste-to-chemicals or waste-to-energy technology that transforms low- or negative-value waste streams into high-value bioproducts such as carboxylic acids, capitalizing on the carboxylate platform. This technology not only bypasses the generation of methane—a typical end product of AD—but also facilitates the production of valuable biochemicals and biofuels through metabolically versatile microbiomes. The strategic inhibition of methanogenesis to favour the production of SCCAs represents an innovative leap in optimizing the valorization of organic waste streams. The involved microbes demonstrate an impressive metabolic flexibility and, depending on their origin, can be adapted to a variety of waste streams for highly efficient MCCA production. Such developments not only promote the sustainability of industrial processes but also enhance economic outcomes by reducing operational costs and increasing the yield of marketable bioproducts. High purity carboxylic acids can be used ‘as is’ or as a platform chemical for producing chemicals and fuels, such as SAF, with a low carbon footprint. Carboxylate separation and purification cost can make up to 64% of total production cost since their separation and purification require energy- and chemical-intensive separation processes. A variety of low-cost and low carbon intensity separation technologies (resins, membrane, and electrochemistry-based technologies) instead of distillation reported promising results, effectively decreasing the separation and purification costs by 75%. However, more work is still needed to bring these technologies to an industrial scale.

Biofilm technologies have introduced a robust platform for waste treatment, particularly in configurations such as dynamic MBRs. These systems exploit biofilms to enhance treatment efficiency and stability, facilitating the recovery of resources from wastewater and supporting the sustainability of water-intensive industries. The application of biofilms in waste treatment not only addresses the technical challenges of high-strength waste streams but also provides a scalable solution that can be integrated into existing infrastructure with minimal disruption. When it comes to understanding the complex structure, function, and regulation of biofilms and microbiomes involved, improved analytical methods are required to develop suitable targets and approaches for engineering the involved microbial consortia. Engineering multi-species biofilms like those present in wastewater systems or microbial conversion systems such as AM and wastewater treatment requires a detailed understanding of the pathways and regulatory system and the inter-species interactions, as well as associated biofilm formation. The energetic valorization of wastewater through advanced biogas technologies such as AnMBRs and the microbial upgrading of biogas to biomethane showcases the integration of waste treatment with energy recovery processes. These technologies enhance the efficiency of organic matter conversion and align with the objectives of reducing greenhouse gas emissions and utilizing renewable energy sources. In addition to improved product separation and purification techniques for carboxylates, this is another critical field for future research.

A particularly innovative aspect covered in this paper is the treatment of recalcitrant waste streams, specifically through an AM process for the biosynthesis of SCCA from marine seaweed—a complex, and underutilized biomass resource. The anaerobic microbial pathways capable of breaking down the seaweed’s complex polysaccharide structure to produce valuable carboxylic acids highlight the potential for tapping into traditionally difficult-to-utilize waste streams. This approach not only enhances the economic viability of marine seaweed as low-cost biomass feedstock but also promotes sustainable waste management.

In conclusion, this comprehensive review underlines a multi-dimensional strategy for waste management that harnesses technological innovations across several domains to transform waste into a resource, using MMC. This paradigm shift not only addresses the immediate challenges posed by increasing waste production and environmental degradation but also aligns with broader sustainability goals. The future of waste management, as suggested by this perspective, lies in the continued development and integration of these technologies into a coherent system that promotes environmental integrity, economic viability, and resource sustainability. As these technologies mature and scale, their integration into a global strategy for waste treatment and resource recovery will be pivotal in achieving a sustainable and circular bioeconomy.

## Data Availability

The data underlying this article are available in the article.
